# Supplementary material for: Non-Convulsive Status Epilepticus in Aneurysmal Subarachnoid Hemorrhage: A Prognostic Parameter
Source: Brain Sci. 2023 Jan 22;13(2):184. doi: 10.3390/brainsci13020184 (PMC9953938; doi:10.3390/brainsci13020184)
Supplement: Supplementary file 1 [file brainsci-13-00184-s001.zip › brainsci-2174784-supplementary.pdf]

Table S1. Aneurysm localization.

Vessel	Number of Patients (n = 506)
Anterior cerebral artery A1	4
Anterior communicating artery	163
Anterior cerebral artery A2	14
Anterior cerebral artery A3–A4	14
Internal carotid artery cavernous segment.	1
Internal carotid artery ophthalmic segment	15
Internal carotid artery terminal segment	83
Internal carotid artery bifurcation	19
Medial cerebral artery M1	5
Medial cerebral artery bifurcation	98
Medial cerebral artery M2	8
Medial cerebral artery M3–M4	2
Basilar artery bifurcation	29
Basilar artery trunk	5
Vertebrobasilar junction	4
Vertebral artery	10
Posterior communicating artery	21
Posterior communicating artery distal segment	5
Posterior cerebral artery	6
